# Linking speciation to extinction: Diversification raises contemporary extinction risk in amphibians

**DOI:** 10.1002/evl3.4

**Published:** 2017-05-03

**Authors:** Dan A. Greenberg, Arne Ø. Mooers

**Affiliations:** ^1^ Department of Biological Sciences Simon Fraser University Burnaby British Columbia V5A 1S6 Canada; ^2^ Earth‐to‐Ocean Research Group Simon Fraser University Burnaby British Columbia V5A 1S6 Canada; ^3^ Crawford Lab for Evolutionary Studies Simon Fraser University Burnaby British Columbia V5A 1S6 Canada

**Keywords:** Amphibia, diversification, extinction risk, IUCN, peripatry, speciation rate, species longevity

## Abstract

Many of the traits associated with elevated rates of speciation, including niche specialization and having small and isolated populations, are similarly linked with an elevated risk of extinction. This suggests that rapidly speciating lineages may also be more extinction prone. Empirical tests of a speciation‐extinction correlation are rare because assessing paleontological extinction rates is difficult. However, the modern biodiversity crisis allows us to observe patterns of extinction in real time, and if this hypothesis is true then we would expect young clades that have recently diversified to have high contemporary extinction risk. Here, we examine evolutionary patterns of modern extinction risk across over 300 genera within one of the most threatened vertebrate classes, the Amphibia. Consistent with predictions, rapidly diversifying amphibian clades also had a greater share of threatened species. Curiously, this pattern is not reflected in other tetrapod classes and may reflect a greater propensity to speciate through peripheral isolation in amphibians, which is partly supported by a negative correlation between diversification rate and mean geographic range size. This clustered threat in rapidly diversifying amphibian genera means that protecting a small number of species can achieve large gains in preserving amphibian phylogenetic diversity. Nonindependence between speciation and extinction rates has many consequences for patterns of biodiversity and how we may choose to conserve it.

**Impact Summary**

The rates of speciation and extinction dictate the frequency at which new species arise and are lost over evolutionary time. Characteristics of species that may promote speciation include being highly specialized to particular environments, existing in isolated populations, or having a low population abundance. These same traits are also associated with extinction: specialized species are vulnerable to environmental change, species that exist in isolated pockets lack population connectivity, and small populations can blink out rapidly. This suggests that lineages speciating readily due to these traits may also readily lose species. Assessing whether speciation and extinction rates are correlated is difficult, as measuring extinction based on fossils can be biased for many groups. However, we are currently in the midst of observing numerous extinctions in real time, and observing variation in the species currently at risk of extinction may serve as a proxy measure for extinction rate across groups. In this study, we show in amphibians that lineages that have high ongoing diversification also have a greater share of species threatened with extinction compared to slowly diversifying groups. This supports the idea that speciation and extinction may go hand‐in‐hand. Comparing this pattern in amphibians to other clades reveals a surprising discrepancy: only plants have been found to show a similar pattern. One mechanism that may produce this link between speciation and extinction could be the mode of speciation–new species arising from isolated populations may be highly specialized, range‐restricted, and vulnerable to extinction. In the grand scheme for amphibian conservation, evolutionarily distinct species are less at risk of extinction—and therefore preserving the amphibian tree of life can be achieved with modest conservation goals. If speciation and extinction rise (and fall) in tandem, this might suggest that lineages may fall along a continuum of producing few, long‐lived species, or many short‐lived species. Linking speciation rates and extinction rates to each other, and to particular modes of speciation, would be an important advance in our understanding of how life on earth diversifies.

## Introduction

The evolutionary rates of speciation and extinction, their difference being diversification rate, shape current patterns of diversity across the tree of life. Standing diversity varies considerably across clades, consistent with lineage‐specific aspects of biology influencing speciation rates, extinction rates, or both (Jablonski 2008). Some biological characteristics that may increase speciation rates include poor dispersal capability (Claramunt et al. 2012), specialization and narrow niche breadths (Rolland and Salamin 2016), large body size (Liow et al. 2008; Monroe and Bokma 2009), or persistence at low population size (Stanley 1990). In turn, these characteristics are also predicted to increase risk of extinction: poor dispersers have limited abilities to (re)colonize or move to suitable environments (Smith and Green 2005; Sandel et al. 2011), specialists are vulnerable to environmental change (McKinney 1997; Colles et al. 2009), large‐bodied species typically have slow life histories (Cardillo et al. 2005; Reynolds et al. 2005), and small populations are subject to demographic stochasticity or extinction from local catastrophies (Lande et al. 2003; Mace et al. 2008). If similar traits drive both speciation and extinction rates, then these rates may be positively correlated across lineages.

Support for a positive speciation‐extinction correlation has remained elusive, in part due to the difficulty of estimating either rate. There is some evidence for a positive speciation‐extinction relationship from the paleontological record in certain groups (Stanley 1990), but for many clades the fossil record is poor. Under certain assumptions, it is possible to estimate speciation and extinction rates separately from phylogenies of extant lineages (Nee et al. 1994), but resultant extinction rates tend to be sorely underestimated (Rabosky 2010). However, we are currently in an era of unprecedented extinction and this unfortunate state of affairs may allow us to directly compare rates of extinction across clades as biodiversity losses accelerate. For certain taxa, clades that seem to have speciated both rapidly and recently have in turn a greater share of currently rare and threatened species (Schwartz and Simberloff 2001; Lozano and Schwartz 2005; Davies et al. 2011), consistent with the expectation under a general speciation‐extinction relationship and suggesting that modern patterns of extinction may serve as a viable surrogate.

Contemporary rates of extinctions are estimated to be magnitudes greater than paleontological rates due to human activities (Pimm et al. 1995; Ceballos et al. 2015). Importantly, although certain drivers of extinction are different in the modern context (Harnik et al. 2012a; Condamine et al. 2013), the same traits associated with modern extinctions have also been linked with species’ lifespan and mass extinctions in the fossil record (McKinney 1997). For instance, geographic range size dominates patterns of modern extinction risk across terrestrial vertebrates (Cardillo et al. 2005; Sodhi et al. 2008; Lee and Jetz 2011; Böhm et al. 2016), and similarly is one of the best predictors of species longevity in the fossil record (Kiessling and Aberhan 2007; Harnik et al. 2012b; Orzechowski et al. 2015; Smits 2015). Specialization has been linked to both modern extinction risk and to species durations in terms of both dietary breadth (Boyles and Storm 2007; Olden et al. 2008; Smits 2015) and habitat/environment breadth (Heim and Peters 2011; Harnik et al. 2012b; Ducatez et al. 2014). Both abundance and body size affect modern extinction risks across taxa (Cardillo et al. 2005; Reynolds et al. 2005; Mace et al. 2008). Fossil evidence also suggests that abundance can dictate the longevity of species (Kiessling and Aberhan 2007), and that large‐bodied species often have higher background and mass extinction rates (Liow et al. 2008; Sallan and Galimberti 2015; but see Smits 2015). If these traits drive both ancient and modern extinctions, and tend to be conserved within lineages over time, then we may expect that extant clades with high contemporary extinction risk should also have high extinction rates over their history. Temporal changes in threats may shift the traits underlying extinction risk (Bromham et al. 2012; Lyons et al. 2016), but many of these traits appear general enough to create consistent long‐term differences in extinction risk (Harnik et al. 2012b; Finnegan et al. 2015; Orzechowski et al. 2015; Smits 2015). Though this concept has yet to be thoroughly tested, emerging evidence suggests that lineages suffering high contemporary extinction risk similarly had high rates of extinction in the fossil record (McKinney 1997; Condamine et al. 2013; Finnegan et al. 2015). Examining modern extinctions may therefore offer an accelerated view of the same patterns that structure paleontological extinction rates across clades.

Net diversification rates are easier to estimate than independent speciation or extinction rates, but diversification is biased towards speciation rates for more recent groups such as genera because extinction must lag speciation (Nee et al. 1994). Therefore diversification rates in extant lineages are often reflective of speciation rates, as is typically inferred through analyses of molecular phylogenies of extant taxa (Rabosky 2010).

If contemporary patterns of extinction reflect paleontological rates, and if diversification rates tend to reflect speciation, then, under the hypothesis of covarying speciation and extinction rates, modern rates of extinction should be positively correlated with diversification rates across young clades. Alternatively, if extinction and speciation are independent then one would expect no correlation between modern rates of extinction and clade diversification rates. Here, we test this hypothesis using patterns of diversification and extinction across 329 genera of Amphibia, a vertebrate group with one of the highest rates of modern extinction (Hoffmann et al. 2010).

## Methods

### TAXONOMIC AND PHYLOGENETIC DATA

We identified amphibian genera that had both phylogenetic and threat status data available that would allow separate estimates of diversification rate and contemporary extinction risk (*N* = 329 genera). We delineated genera based on the taxonomy from the Amphibian Species of the World database v6.0 (Frost 2016) and included all monophyletic clades that (i) had at least one species assessed for threat status by the International Union for the Conservation of Nature (IUCN) Red List (IUCN 2016), (ii) that had both crown and stem group ages, and (iii) that had more than two representatives on the phylogeny for non‐mono/ditypic genera (to mitigate against underestimating crown ages). For each genus we compiled data on extant species richness, and both crown and stem group age. Extant species richness (n) was assessed based on species counts in the Amphibian Species of the World database. Crown and stem group ages (in millions of years) were estimated from one of the most extensive published, time‐calibrated phylogenies for amphibians (Pyron 2014). Net diversification rates can be estimated either by crown or stem ages (Magallon and Sanderson 2001). Both estimators have their drawbacks: crown ages exclude monotypic genera, and stem ages are shared between pairs of lineages. We therefore considered both stem and crown diversification‐rates using the method‐of‐moments estimator (Magallon and Sanderson 2001).

### EXTINCTION RISK

To characterize the contemporary extinction rate for each clade, we assessed the proportion of species in each genus that are currently threatened with extinction. Each amphibian species that has been assessed by the IUCN Red List (*n* = 6460; IUCN 2016) was classified based on their threat category as either “threatened” (IUCN threat categories: Vulnerable (VU), Endangered (EN), Critically Endangered (CR), Extinct in the Wild (EW), or Extinct (EX)) or “nonthreatened” (species listed as Least Concern (LC) and Near‐Threatened (NT)). For each genus our measure of extinction rate was the proportion of “threatened” species.

### RANGE SIZE PATTERNS

Geographic range size is typically the dominant driver of extinction risk for terrestrial vertebrates (Cardillo et al. 2005; Sodhi et al. 2008; Lee and Jetz 2011), and evolutionary processes can shape patterns of geographic distributions considerably (Barraclough and Vogler 2000). Species range‐restriction has also been associated with heightened rates of speciation in some taxa (Jablonski and Roy 2003; Price and Wagner 2004), including certain groups of amphibians (Eastman and Storfer 2011; Wollenberg et al. 2011). To investigate whether relationships between extinction risk and diversification might be mediated through species’ range size patterns we examined associations between genera diversification rate and the mean logarithmic extent of occurrence across species. Range size, in km^2^_,_ was estimated for 6311 species based on extent of occurrence polygons from the IUCN (IUCN 2016).

### ANALYSIS

To determine the role of evolutionary diversification on contemporary patterns of extinction across genera we used phylogenetic generalized linear models, which can control for phylogenetic autocorrelation in extinction risk across genera. Extinction risk (proportion of threatened species per genus) was fit with a binomial error distribution. Models were run using uninformative priors for 2 × 10^6^ generations with a 2 × 10^5^ burn‐in, and a sampling interval of 1000. We compared models examining the relationship between proportion of species threatened per genus and species richness, crown and stem age, and diversification rate based on stem or crown ages. Species richness and lineage ages were log_e_ transformed, and crown diversification rate was square root transformed, to improve their distributions. To describe the relationship between mean species’ range size (log_e_ transformed) and diversification rate across genera, we used the same modeling approach with a Gaussian error distribution. The significance of richness, age, and diversification were evaluated based on the 95% credibility intervals (CI) of the coefficient estimates. We calculated the mean correlation coefficient (*r*) between predicted and observed genus extinction risk to evaluate the fit for each model. Analyses were performed using the package “MCMCglmm” (Hadfield 2010) in R v. 3.3.3.

## Results

**Figure 1:**
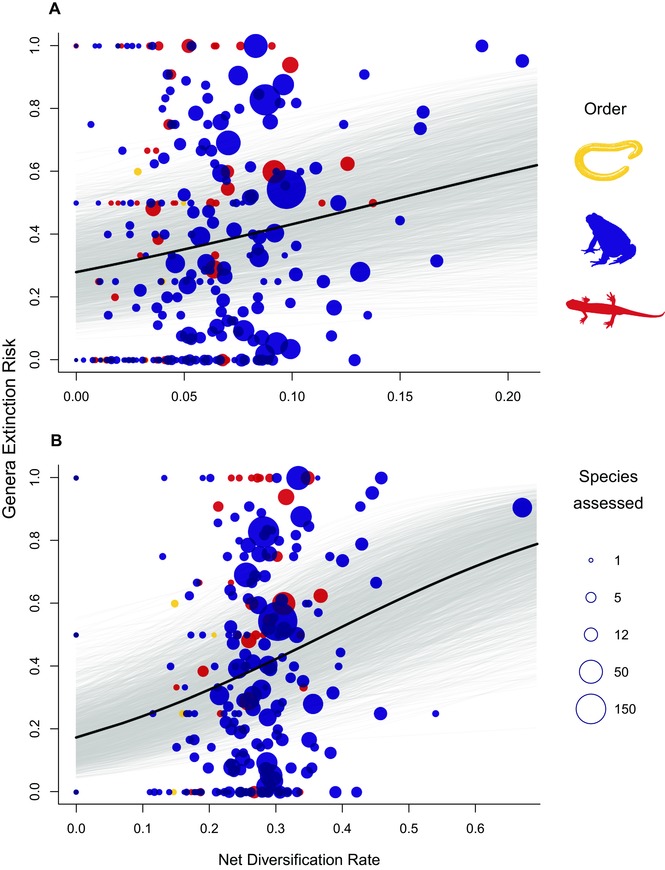
Plot of the proportion of globally threatened species and diversification rate across amphibian genera, showing a positive relationship between extinction risk (proportion species threatened) and net diversification rate calculated using (A) stem age (*n* = 329) and (B) crown group age (square root transformed, *n* = 247). Gray lines indicate the fitted relationships (1800 samples) drawn from the posterior distribution of the models.

Extinction risk was distributed unevenly across the amphibian genera, with rapidly diversifying clades having a greater share of threatened species (Fig. 1); this holds true for diversification rates estimated from both stem ages (β = 7.55, 95% CI = 1.32, 14.66, *p*MCMC = 0.02, Fig. 1A) and crown ages (β = 4.16, 95% CI = 1.70, 6.53, *p*MCMC < 0.001, Fig. 1B; these two diversification estimates were moderately correlated, *r* = 0.69). Diversification rate (for both stem and crown group age) was the best evolutionary descriptor of the distribution of threat across these clades, as neither species richness, stem age, nor crown age had a significant influence on extinction risk (Table 1).

**Table 1:** Summary of generalized linear models relating log_e_ (genera species richness), log_e_ (age), and net diversification rate (square root transformed for crown diversification rate) to patterns of extinction risk (proportion of threatened species) for all genera with stem ages (top, including monotypic genera, *n* = 329) and all genera with crown ages (bottom, *n* = 247)

Variable	β (95% CI)	*p*MCMC	Pagel's λ (95% CI)	*r*
Species richness	0.108 (−0.08, 0.26)	0.201	0.43 (0.33, 0.51)	0.047
Stem age	−0.345 (−0.83, 0.16)	0.170	0.44 (0.35, 0.51)	0.081
Stem diversification rate	6.735 (0.91, 12.61)	0.018	0.43 (0.35, 0.51)	0.087
Species richness	0.189 (−0.04, 0.39)	0.094	0.42 (0.33, 0.50)	0.097
Crown age	−0.339 (−0.76, 0.06)	0.120	0.43 (0.33, 0.51)	0.083
Crown diversification rate	4.162 (1.70, 6.53)	< 0.001	0.43 (0.33, 0.51)	0.178

Coefficients represent the posterior mean and correspond to a logit link, and *r* represents the correlation between observed and model predicted genus extinction risk.

Considering only the subset of genera that have both crown and stem diversification rates (and so have at least two species) we found that the relationship between contemporary extinction risk and stem diversification was even stronger in this subset (β = 11.42, 95% CI = 3.96, 21.02; *p*MCMC = 0.01), suggesting that monotypic genera may contribute to uncertainty in the pattern. Although explanatory power was generally modest (Table 1), the models are robust: the proportion of threatened species significantly increases with crown diversification rate when removing when removing both the most rapidly diversifying, and highly threatened, clade *Telmatobius* (β = 3.78, 95% CI = 1.52, 6.40, *p*MCMC = 0.001), and also when removing the 10% highest diversifying clades (*n* = 224; β = 3.41, 95% CI = 0.49, 6.27, *p*MCMC = 0.026).

**Figure 2:**
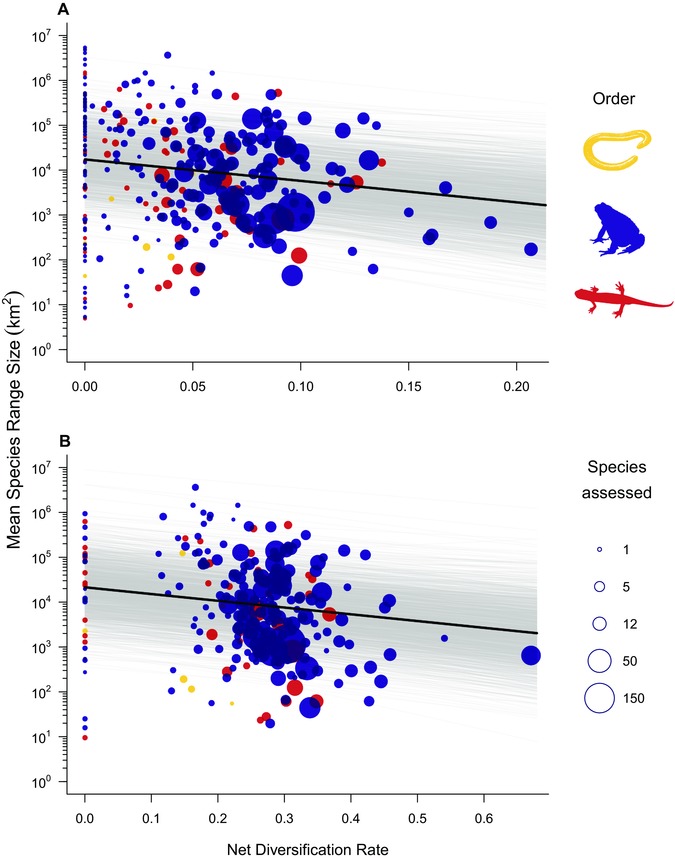
Plot of mean species’ geographic range size (km^2^) and net diversification rate across amphibian genera, calculated using (A) stem age and (B) crown group age (square root transformed). Gray lines indicate the fitted relationships (1800 samples) from the posterior distribution of the models.

Across these 329 genera there was a strong phylogenetic signal in average species’ range size (Pagel's λ = 0.73, 95% CI = 0.54, 0.82), and in addition to having a greater share of threatened species, rapidly diversifying genera also contained species with smaller mean geographic ranges (β = –11.00, 95% CI = –3.08, –18.24, *p*MCMC = 0.004, Fig. 2A; β = –3.47, 95% CI = –0.79, –6.18, *p*MCMC = 0.01, Fig. 2B).

## Discussion

The positive relationship between the proportion of currently threatened species and their evolutionary diversification across amphibian genera is consistent with theory linking speciation and extinction rates across clades. Importantly, diversification rate had a much stronger influence than lineage age or species richness, suggesting that the process of speciation itself could be driving this relationship.

The causal mechanisms expected to simultaneously drive speciation and extinction rates are general across biodiversity (Stanley 1990), suggesting that this pattern should be widespread. Although evidence of a positive correlation between these rates has been found in fossil data among different groups (Stanley 1979; Jablonski 1986; Gilinsky 1994; Liow et al. 2008), there appears to be little support for a link between diversification and modern extinction risk across other vertebrates. Neither birds (Jetz et al. 2014), nor mammals (Verde Arregoitia et al. 2013), nor squamate reptiles (Tonini et al. 2016), exhibit any association between evolutionary distinctiveness (a species‐level measure of diversification; Jetz et al. 2012) and threat status. The only other group where a direct link between diversification and extinction risk has been demonstrated is within angiosperms from the Cape of South Africa (Davies et al. 2011). In this highly endemic region, the youngest, rapidly diversifying clades also have a greater share of threatened species. This pattern of heightened extinction risk in diversifying plant clades may be a general phenomenon, as species rarity rises in tandem with clade richness in vascular plants across both taxonomic levels and geographic realms (Schwartz and Simberloff 2001; Lozano and Schwartz 2005). This raises a key question: what do amphibians have more in common with plants than with their tetrapod counterparts?

A pattern of positively correlated speciation and extinction may ultimately be driven by mode of speciation. Amphibians often have specialized breeding habitat requirements and are generally poor dispersers (Smith and Green 2005; Wells 2007), which may produce many small, geographically isolated populations that in turn encourage speciation. This form of peripatric speciation may predominate for amphibians, as has been suggested for plant speciations in South Africa and observed in the heightened rates of species rarity in speciose plant families (Schwartz and Simberloff 2001; Lozano and Schwartz 2005; Davies et al. 2011). Under this hypothesis, rapidly speciating clades would produce a preponderance of range‐restricted species that are in turn highly threatened by anthropogenic drivers (Sodhi et al. 2008). Indeed, we found that genera diversification rate was negatively correlated with average species’ range size, consistent with peripatry being a potential mechanism driving an association between speciation and extinction. Alternatively, it may not be that peripatric speciation dominates in amphibians, but rather that some other biological trait both drives diversification and tends to limit range size, for example small body size or narrow niche breadths (Wollenberg et al. 2011; Slatyer et al. 2013). We might also expect that species’ geography, and its heritability, could play an important role driving both speciation and extinction across clades if certain physical environments or biomes concurrently drive both processes. Understanding how the form and tempos of speciation relates to species’ characteristics will be critical to unraveling these evolutionary patterns of extinction in the amphibians.

Another compelling question concerns how these patterns of impending extinction might shape the future amphibian tree of life. We can estimate the expected loss of phylogenetic diversity based on current patterns of extinction risk: if all currently threatened species were lost across the 329 genera in our dataset, then we would lose 21.55% of genus‐level phylogenetic diversity. However, an even distribution of threat across these same genera would result in significantly less phylogenetic diversity loss at 20.05% (95% CI = 19.0%, 21.1%; see electronic supplementary material). This runs counter to the typical expectation for the loss of evolutionary history when speciation and extinction are positively correlated (Heard and Mooers 2000; but see Parhar and Mooers 2011). Interestingly, our result is due to a subset of clades facing complete lineage extinction, in that all species are threatened. Saving just one species, irrespective of identity, in each of these genera (*n* = 20) would prevent the loss of an estimated 1.4 billion years of evolutionary history. From this perspective the most effective method to preserve amphibian biodiversity in an age of contemporary mass extinction may entail shifting some focus from species to lineages, even if this means allowing some extinction of phylogenetically redundant species in rapidly diversifying lineages.

A link between speciation and extinction rates has many consequences for shaping past, present, and future patterns of biodiversity. It may suggest that lineages fall along a slow‐to‐fast continuum for species turnover, where rapidly speciating lineages produce short‐lived, extinction‐prone species due to shared traits driving both speciation and extinction processes in tandem (Stanley 1990). There is some limited evidence for this including patterns of higher species turnover in large‐bodied mammals (Liow et al. 2008; Monroe and Bolkma 2009), that speciose plant clades may both produce and lose many rare species (Schwartz and Simberloff 2001; Davies et al. 2011), the reduced species longevity and heightened origination of range‐restricted marine gastropods (Jablonski 1986), and the elevated speciation and extinction rates of specialist taxa generally (Colles et al. 2009; Rolland and Salamin 2016). The lack of association between evolutionary distinctiveness and threat among birds (Jetz et al. 2014), mammals (Verde Arregoitia et al. 2013), and reptiles (Tonini et al. 2016), may indicate that either these patterns do not arise at the taxonomic scale of species or that high clade turnover obscures the relationship between net diversification and extinction risk in these groups. Analyzing this same question at the species‐level for amphibians might help resolve this paradox and, importantly, account for other processes driving contemporary extinction risk that may have contributed to the fairly low explanatory power of diversification at the genus level. For instance, a species‐level analysis would allow us to assess the role of geography in patterns of diversification and extinction in amphibians (see, e.g., Pyron and Wiens 2013). However, this crucial step is currently precluded by the lack of a fully sampled amphibian phylogeny necessary for such an analysis. To account for turnover, independently estimating speciation and extinction rates, perhaps through combining both fossil and molecular phylogenetic data in well‐sampled clades, will be key to assess whether speciation and extinction rates are concurrently driven by biological characteristics across a diverse set of taxa.

Ecological limits may also be crucial to a positive speciation‐extinction correlation. Clades near their carrying capacity, where speciation and extinction balance out, may be expected to exhibit the positive relationship we report here, while clades in their diversity “growth phase” may be able to escape this trade‐off (Rabosky 2009; Etienne et al. 2012). This growth phase may be associated with novel ecological opportunities or adaptations that may allow some high turnover clades to temporarily decouple speciation and extinction rates and undergo adaptive radiations (Rabosky and Lovette 2008). Understanding the conditions that maintain, or break down, any relationship between speciation and extinction rates will be key to our understanding of the long‐term temporal dynamics of biodiversity.

Here, we demonstrate that net diversification is associated with a greater contemporary extinction risk across amphibian genera. This pattern is consistent with the theory that speciation and extinction rates may be driven by the same suites of traits, or by common geography, resulting in clades that both rapidly diversify and lose species. Nonindependence of speciation and extinction rates would add a new piece to both understanding temporal patterns of biodiversity and how we may aim to prioritize and manage that biodiversity in the present.

## Supporting information


**Table S1**. Twenty amphibian genera with all assessed species at risk, and therefore facing potential lineage extinction, indicating the species richness and phylogenetic diversity (PD) within each group, the lineage's stem age, and the potential prevention of PD loss by saving one species within each clade (PD saved).

Supporting Information

## References

[evl34-bib-0001] Barraclough, T. G. , and A. P. Vogler . 2000 Detecting the geographical pattern of speciation from species‐level phylogenies. Am. Nat. 155:419–434. 10753072 10.1086/303332

[evl34-bib-0002] Böhm, M. , R. Williams , H. R. Bramhall , K. M. McMillan , A. D. Davidson , A. Garcia , L. M. Bland , J. Bielby , and B. Collen . 2016 Correlates of extinction risk in squamate reptiles: the relative importance of biology, geography, threat and range size. Glob. Ecol. Biogeogr. 25:391–405.

[evl34-bib-0003] Boyles, J. G. , and J. J. Storm . 2007 The perils of picky eating: dietary breadth is related to extinction risk in insectivorous bats. PLoS One 2:e672. 17653286 10.1371/journal.pone.0000672 PMC1914379

[evl34-bib-0004] Bromham, L. , R. Lanfear , P. Cassey , G. Gibb , and M. Cardillo . 2012 Reconstructing past species assemblages reveals the changing patterns and drivers of extinction through time. Proc. R. Soc. Lond. B 279:4024–4032. 10.1098/rspb.2012.1437 PMC3427590 22859591

[evl34-bib-0005] Cardillo, M. , G. M. Mace , K. E. Jones , J. Bielby , O. R. P. Bininda‐Emonds , W. Sechrest , *et al* 2005 Multiple causes of high extinction risk in large mammal species. Science 309:1239–1241. 16037416 10.1126/science.1116030

[evl34-bib-0006] Ceballos, G. , P. R. Ehrlich , A. D. Barnosky , A. García , R. M. Pringle , and T. M. Palmer . 2015 Accelerated modern human–induced species losses: entering the sixth mass extinction. Sci. Adv. 1:e1400253. 26601195 10.1126/sciadv.1400253 PMC4640606

[evl34-bib-0007] Claramunt, S. , E. P. Derryberry , J. V. Remsen , and R. T. Brumfield . 2012 High dispersal ability inhibits speciation in a continental radiation of passerine birds. Proc. R. Soc. Lond. B 279:1567–1574. 10.1098/rspb.2011.1922 PMC3282344 22090382

[evl34-bib-0008] Colles, A. , L. H. Liow , and A. Prinzing . 2009 Are specialists at risk under environmental change? Neoecological, paleoecological and phylogenetic approaches. Ecol. Lett. 12:849–863. 19580588 10.1111/j.1461-0248.2009.01336.x PMC2730552

[evl34-bib-0009] Condamine, F. L. , J. Rolland , and H. Morlon 2013 Macroevolutionary perspectives to environmental change. Ecol. Lett. 16:72–85. 10.1111/ele.12062 23331627

[evl34-bib-0010] Davies, T. J. , G. F. Smith , D. U. Bellstedt , J. S. Boatwright , B. Bytebier , R. M. Cowling , F. Forest , L. J. Harmon , A. M. Muasya , B. D. Schrire , *et al* 2011 Extinction risk and diversification are linked in a plant biodiversity hotspot. PLoS Biol. 9:e1000620. 21629678 10.1371/journal.pbio.1000620 PMC3101198

[evl34-bib-0011] Ducatez, S. , R. Tingley , and R. Shine . 2014 Using species co‐occurrence patterns to quantify relative habitat breadth in terrestrial vertebrates. Ecosphere 5:art152.

[evl34-bib-0012] Eastman, J. M. , and A. Storfer 2011 Correlations of life‐history and distributional‐range variation with salamander diversification rates: evidence for species selection. Syst. Biol. 60:503–518. 21460386 10.1093/sysbio/syr020

[evl34-bib-0013] Etienne, R. S. , B. Haegeman , T. Stadler , T. Aze , P. N. Pearson , A. Purvis , and A. B. Philimore . 2012 Diversity‐dependence brings molecular phylogenies closer to agreement with the fossil record. Proc. R. Soc. Lond. B 279:1300–1309. 10.1098/rspb.2011.1439 PMC3282358 21993508

[evl34-bib-0014] Finnegan, S. , S. C. Anderson , P. G. Harnik , C. Simpson , D. P. Tittensor , J. E. Byrnes , Z. V. Finkel , D. R. Lindberg , L. H. Liow , R. Lockwood , *et al* 2015 Paleontological baselines for evaluating extinction risk in the modern oceans. Science 348:567–570. 25931558 10.1126/science.aaa6635

[evl34-bib-0015] Frost, D. R. 2016 Amphibian Species of the World: An Online Reference. Version 6.0. Available at: http://research.amnh.org/vz/herpetology/amphibia.

[evl34-bib-0016] Gilinsky, N. L. 1994 Volatility and the Phanerozoic decline of background extinction intensity. Paleobiology 20:445–458.

[evl34-bib-0017] Hadfield, J. 2010 MCMC methods for multi‐response generalized linear mixed models: the MCMCglmm R package. J. Stat. Softw. 33:1–22.20808728

[evl34-bib-0018] Harnik, P. G. , H. K. Lotze , S. C. Anderson , Z. V. Finkel , S. Finnegan , D. R. Lindberg , *et al* 2012a Extinctions in ancient and modern seas. Trends Ecol. Evol. 27:608–617. 22889500 10.1016/j.tree.2012.07.010

[evl34-bib-0019] Harnik, P. G. , C. Simpson , and J. L. Payne . 2012b Long‐term differences in extinction risk among the seven forms of rarity. Proc. R. Soc. Lond. B 279:4969–4976. 10.1098/rspb.2012.1902 PMC3497235 23097507

[evl34-bib-0020] Heard, S. B. , and A. Ø. Mooers . 2000 Phylogenetically patterned speciation rates and extinction risks change the loss of evolutionary history during extinctions. Proc. R. Soc. Lond. B 267:613–620. 10.1098/rspb.2000.1046 PMC1690578 10787167

[evl34-bib-0021] Heim, N. A. , and S. E. Peters . 2011 Regional environmental breadth predicts geographic range and longevity in fossil marine genera. PLoS One 6:e18946. 21573226 10.1371/journal.pone.0018946 PMC3087726

[evl34-bib-0022] Hoffmann, M. , C. Hilton‐Taylor , A. Angulo , M. Böhm , T. M. Brooks , S. H. M. Butchart , K. E. Carpenter , J. Chanson , B. Collen , N. A. Cox , *et al* 2010 The impact of conservation on the status of the world's vertebrates. Science 330:1503–1509. 20978281 10.1126/science.1194442

[evl34-bib-0023] IUCN . 2016 The IUCN Red List of Threatened Species. Version 2016‐1. Available at: http://www.iucnredlist.org

[evl34-bib-0024] Jablonski, D. 1986 Larval ecology and macroevolution in marine invertebrates. Bull. Mar. Sci. 39:565–587.

[evl34-bib-0025] Jablonski, D. 2008 Species selection: theory and data. Annu. Rev. Ecol. Evol. Syst. 39:501–524.

[evl34-bib-0026] Jablonski, D. , and K. Roy . 2003 Geographical range and speciation in fossil and living molluscs. Proc. R. Soc. Lond. B 270:401–406. 10.1098/rspb.2002.2243 PMC1691247 12639320

[evl34-bib-0027] Jetz, W. , G. H. Thomas , J. B. Joy , K. Hartmann , and A. Ø. Mooers . 2012 The global diversity of birds in space and time. Nature 491:444–448. 23123857 10.1038/nature11631

[evl34-bib-0028] Jetz, W. , G. H. Thomas , J. B. Joy , D. W. Redding , K. Hartmann , and A. Ø. Mooers . 2014 Global distribution and conservation of evolutionary distinctness in birds. Curr. Biol. 24:919–930. 24726155 10.1016/j.cub.2014.03.011

[evl34-bib-0029] Kiessling, W. , and M. Aberhan . 2007 Geographical distribution and extinction risk: lessons from Triassic‐Jurassic marine benthic organisms. J. Biogeogr. 34:1473–1489.

[evl34-bib-0030] Lande, R. , S. Engen , and B.‐E. Sæther . 2003 Stochastic Population Dynamics in Ecology and Conservation. Oxford Univ. Press, Oxford, UK.

[evl34-bib-0031] Lee, T. M. , and W. Jetz . 2011 Unravelling the structure of species extinction risk for predictive conservation science. Proc. R. Soc. Lond. B 278:1329–1338. 10.1098/rspb.2010.1877 PMC3061137 20943690

[evl34-bib-0032] Liow, L. H. , M. Fortelius , E. Bingham , K. Lintulaakso , H. Mannila , L. Flynn , and N. C. Stenseth . 2008 Higher origination and extinction rates in larger mammals. Proc. Natl. Acad. Sci. USA 105:6097–6102. 18417455 10.1073/pnas.0709763105 PMC2329699

[evl34-bib-0033] Lozano, F. D. , and M. W. Schwartz . 2005 Patterns of rarity and taxonomic group size in plants. Biol. Conserv. 126:146–154.

[evl34-bib-0034] Lyons, S. K. , J. H. Miller , D. Fraser , F. A. Smith , A. Boyer , E. Lindsey , and A. M. Mychajliw . 2016 The changing role of mammal life histories in Late Quaternary extinction vulnerability on continents and islands. Biol. Lett. 12:20160342. 27330176 10.1098/rsbl.2016.0342 PMC4938058

[evl34-bib-0035] Mace, G. M. , N. J. Collar , K. J. Gaston , C. Hilton‐Taylor , H. R. Akçakaya , N. Leader‐Williams , E. J. Milner‐Gulland , and S. N. Stuart . 2008 Quantification of extinction risk: IUCN's system for classifying threatened species. Conserv. Biol. 22:1424–1442. 18847444 10.1111/j.1523-1739.2008.01044.x

[evl34-bib-0036] Magallon, S. , and M. J. Sanderson . 2001 Absolute diversification rates in Angiosperm clades. Evolution 55:1762–1780. 11681732 10.1111/j.0014-3820.2001.tb00826.x

[evl34-bib-0037] McKinney, M. L. 1997 Extinction vulnerability and selectivity: combining ecological and paleontological views. Annu. Rev. Ecol. Syst. 28:495–516.

[evl34-bib-0038] Monroe, M. J. , and F. Bokma . 2009 Do speciation rates drive rates of body size evolution in mammals? Am. Nat. 174:912–918. 19860548 10.1086/646606

[evl34-bib-0039] Nee, S. , E. C. Holmes , R. M. May , and P. H. Harvey . 1994 Extinction rates can be estimated from molecular phylogenies. Philos. Trans. R. Soc. Lond. B. Biol. Sci. 344:77–82. 8878259 10.1098/rstb.1994.0054

[evl34-bib-0040] Olden, J. D. , N. L. Poff , and K. R. Bestgen . 2008 Trait synergisms and the rarity, extirpation, and extinction risk of desert fishes. Ecology 89:847–856. 18459347 10.1890/06-1864.1

[evl34-bib-0041] Orzechowski, E. A. , R. Lockwood , J. E. K. Byrnes , S. C. Anderson , S. Finnegan , Z. V. Finkel , P. G. Harnik , D. R. Lindberg , L. H. Liow , H. K. Lotze , *et al* 2015 Marine extinction risk shaped by trait‐environment interactions over 500 million years. Glob. Chang. Biol. 21:3595–3607. 26190141 10.1111/gcb.12963

[evl34-bib-0042] Parhar, R. K. , and A. Ø. Mooers . 2011 Phylogenetically clustered extinction risks do not substantially prune the Tree of Life. PLoS One 6:e23528. 21853147 10.1371/journal.pone.0023528 PMC3154466

[evl34-bib-0043] Pimm, S. L. , G. J. Russell , J. L. Gittleman , and T. M. Brooks . 1995 The future of biodiversity. Science 269:347–350. 17841251 10.1126/science.269.5222.347

[evl34-bib-0044] Price, J. P. , and W. L. Wagner . 2004 Speciation in Hawaiian angiosperm lineages: cause, consequence, and mode. Evolution 58:2185–2200. 15562684 10.1111/j.0014-3820.2004.tb01597.x

[evl34-bib-0045] Pyron, R. A. 2014 Biogeographic analysis reveals ancient continental vicariance and recent oceanic dispersal in amphibians. Syst. Biol. 63: 779–797. 24951557 10.1093/sysbio/syu042

[evl34-bib-0046] Pyron, R. A. , and J. J. Wiens . 2013 Large‐scale phylogenetic analyses reveal the causes of high tropical amphibian diversity. Proc. R. Soc. Lond. 280:20131622. 10.1098/rspb.2013.1622 PMC3779328 24026818

[evl34-bib-0047] Rabosky, D. L. 2009 Ecological limits and diversification rate: alternative paradigms to explain the variation in species richness among clades and regions. Ecol. Lett. 12:735–743. 19558515 10.1111/j.1461-0248.2009.01333.x

[evl34-bib-0048] Rabosky, D. L. 2010 Extinction rates should not be estimated from molecular phylogenies. Evolution 64:1816–1824. 20030708 10.1111/j.1558-5646.2009.00926.x

[evl34-bib-0049] Rabosky, D. L. , and I. J. Lovette . 2008 Explosive evolutionary radiations: decreasing speciation or increasing extinction through time? Evolution 62:1866–1875. 18452577 10.1111/j.1558-5646.2008.00409.x

[evl34-bib-0050] Reynolds, J. D. , N. K. Dulvy , N. B. Goodwin , and J. A. Hutchings . 2005 Biology of extinction risk in marine fishes. Proc. R. Soc. Lond. B 272:2337–2344. 10.1098/rspb.2005.3281 PMC1559959 16243696

[evl34-bib-0051] Rolland, J. , and N. Salamin . 2016 Niche width impacts vertebrate diversification. Glob. Ecol. Biogeogr. 25:1252–1263.

[evl34-bib-0052] Sallan, L. , and A. K. Galimberti . 2015 Body‐size reduction in vertebrates following the end‐Devonian mass extinction. Science 350:812–815. 26564854 10.1126/science.aac7373

[evl34-bib-0053] Sandel, B. , L. Arge , B. Dalsgaard , R. G. Davies , K. J. Gaston , W. J. Sutherland , and J.‐C. Svenning . 2011 The influence of Late Quaternary climate‐change velocity on species endemism. Science 334:660–664. 21979937 10.1126/science.1210173

[evl34-bib-0054] Schwartz, M. W. , and D. Simberloff . 2001 Taxon size predicts rates of rarity in vascular plants. Ecol. Lett. 4:464–469.

[evl34-bib-0055] Slatyer, R. A. , M. Hirst , and J. P. Sexton . 2013 Niche breadth predicts geographical range size: a general ecological pattern. Ecol. Lett. 16:1104–1114. 23773417 10.1111/ele.12140

[evl34-bib-0056] Smith, A. M. , and D. M. Green . 2005 Dispersal and the metapopulation paradigm in amphibian ecology and conservation: are all amphibian populations metapopulations? Ecography 28:110–128.

[evl34-bib-0057] Smits, P. D. 2015 Expected time‐invariant effects of biological traits on mammal species duration. Proc. Natl. Acad. Sci. USA 112:13015–13020. 26438873 10.1073/pnas.1510482112 PMC4620871

[evl34-bib-0058] Sodhi, N. S. , D. Bickford , A. C. Diesmos , T. M. Lee , L. P. Koh , B. W. Brook , C. H. Sekercioglu , and C. J. A. Bradshaw . 2008 Measuring the meltdown: Drivers of global amphibian extinction and decline. PLoS One 3:e1636. 18286193 10.1371/journal.pone.0001636 PMC2238793

[evl34-bib-0059] Stanley, S. M. 1979 Macroevolution, pattern and process. W. H. Freeman, San Francisco, USA.

[evl34-bib-0060] Stanley, S. M. 1990 The general correlation between rate of speciation and rate of extinction: fortuitous causal linkages Pp. 103–172 *in* Ross R. M. and Allmon W. D., eds. Causes of Evolution: A Paleontological Perspective. Chicago Univ. Press, Chicago, USA.

[evl34-bib-0061] Tonini, J. F. R. , K. H. Beard , R. B. Ferreira , W. Jetz , and R. A. Pyron . 2016 Fully‐sampled phylogenies of squamates reveal evolutionary patterns in threat status. Biol. Conserv. 204:23–31.

[evl34-bib-0062] Verde Arregoitia, L. D. , S. P. Blomberg , and D. O. Fisher . 2013 Phylogenetic correlates of extinction risk in mammals: species in older lineages are not at greater risk. Proc. R. Soc. Lond. B 280:20131092. 10.1098/rspb.2013.1092 PMC3712450 23825210

[evl34-bib-0063] Wells, K. D. 2007 The ecology and behavior of amphibians. Chicago Univ. Press, Chicago, USA.

[evl34-bib-0064] Wollenberg, K. C. , D. R. Vieites , F. Glaw , and M. Vences . 2011 Speciation in little: the role of range and body size in the diversification of *Malagasy mantellid* frogs. BMC Evol. Biol. 11:217. 21777445 10.1186/1471-2148-11-217 PMC3199771

