# Supplementary material for: Linking speciation to extinction: Diversification raises contemporary extinction risk in amphibians
Source: Evol Lett. 2017 May 3;1(1):40–8. doi: 10.1002/evl3.4 (PMC6121784; doi:10.1002/evl3.4)

**Supplementary Material**

*Estimates of Phylogenetic Diversity Loss*

To determine how the observed distribution of threat influences the expected loss of phylogenetic diversity across all 329 amphibian genera compared to an even distribution of threat, we ran 2000 simulations where each species had a 44.61% probability of being lost (the overall proportion of threatened species in the dataset). The phylogenetic diversity per genus was estimated based on the genus’ net diversification rate using the crown age method-of-moment estimator according to equation in Theorem 5 from Mooers et al. 2012:

$Clade Phylogenetic Diversity= \frac{2*e^{R*t}}{\mu}*ln(\frac{\rho*e^{R*t}-1}{\left( \rho-1 \right)*e^{R*t}})$ (1)

Whereby *R* is the net diversification rate, *t* is the crown age, *μ* is the assumed extinction rate, and ρ is the relative speciation rate (λ/μ). For estimates of genera phylogenetic diversity we assumed a value of ρ = 1.49. We used either the clade stem age or double the crown age as the estimate of phylogenetic diversity for monotypic genera and ditypic genera, respectively. The total phylogenetic diversity represented by all genera included both their within clade phylogenetic diversity (eq. 1) and the clades’ stem ages.

The surviving phylogenetic diversity in each clade following pruning of threatened species was based on the observed extinction risk in each genera, using equation 7 from Mooers et al. 2012:

$s=1-\frac{N_{threatened}}{N_{assessed}}$ (2)

$a=1-\frac{1}{\rho}$ (3)

$Surviving Clade Phylogenetic Diversity= \frac{s}{(s-a)}*ln( \frac{s}{a})*\frac{1-a}{\ln(a)}$ (4)

The estimated phylogenetic diversity loss was the difference between surviving phylogenetic diversity for each genera relative to total clade phylogenetic diversity. When a clade was facing complete extinction, ie. all species are threatened, then the clades’ stem was considered to be lost as well and this was counted towards loss of total phylogenetic diversity across the 329 genera.

Simulating random extinctions across the 329 genera in proportion to their extant diversity would, on average, represent an estimated loss of 10,605 million years of evolutionary history (95% confidence intervals: 10,050 m.y., 11,181 m.y.). If each genus suffered extinction proportional to its current ratio of ‘threatened’-to-‘non-threatened’ species, then a total of 11,371 million years of evolutionary history would be lost. For comparison, the species in these 329 genera are estimated to represent ~52,884 million years of evolutionary history in total.

To investigate patterns of phylogenetic diversity loss, we identified all non-monotypic clades that are threatened with complete extinction (ie. all species are threatened). We altered the extinction risk of each clade (n = 20; Table S1) by reducing the number of threatened species by 1, and recalculated the surviving phylogenetic diversity in each clade and the additional effect of saving each stem. This resulted in an estimated total loss of 9967 m.y. of evolutionary history, a difference of 1.4 billion years from the previous estimate based on the observed proportions.

**Table S1.** Twenty amphibian genera with all assessed species at risk, and therefore facing potential lineage extinction, indicating the species richness and phylogenetic diversity (PD) within each group, the lineage’s stem age, and the potential prevention of PD loss by saving one species within each clade (PD saved).

| **Genus** | **Genus richness** | **Stem age** | **Estimated PD** | **PD saved** |
| --- | --- | --- | --- | --- |
| *Atelopus* | 96 | 54.90743 | 463.7012 | 88.6121 |
| *Barbourula* | 2 | 64.68169 | 56.07272 | 106.1026 |
| *Batrachuperus* | 6 | 19.77388 | 33.06141 | 35.17404 |
| *Celsiella* | 2 | 20.37109 | 26.09523 | 46.46633 |
| *Cryptotriton* | 7 | 57.20819 | 44.42109 | 80.50812 |
| *Dendrotriton* | 8 | 54.3852 | 49.06105 | 71.94602 |
| *Hoplophryne* | 2 | 78.76856 | 46.04282 | 112.7804 |
| *Isthmura* | 6 | 27.66576 | 46.47246 | 52.04169 |
| *Ixalotriton* | 2 | 33.03582 | 28.53986 | 54.11821 |
| *Liuixalus* | 7 | 67.22534 | 23.42124 | 84.52661 |
| *Lyciasalamandra* | 10 | 30.25005 | 56.12362 | 51.93925 |
| *Lynchius* | 4 | 58.80917 | 78.68933 | 106.6179 |
| *Neurergus* | 4 | 21.68313 | 22.95125 | 33.72161 |
| *Phrynopus* | 28 | 62.3764 | 160.47 | 112.7496 |
| *Plectrohyla* | 18 | 15.38757 | 43.41713 | 24.85855 |
| *Probreviceps* | 6 | 50.81095 | 62.11515 | 76.98978 |
| *Sechellophryne* | 2 | 35.83857 | 39.02736 | 64.66807 |
| *Sooglossus* | 2 | 35.83857 | 35.09326 | 61.76195 |
| *Taruga* | 3 | 42.32515 | 21.1003 | 55.1449 |
| *Thorius* | 26 | 62.62314 | 111.5563 | 83.37591 |

**References**

Mooers, A. Ø., Gascuel, O., Stadler, T., Li, H. & Steel, M. 2012 Branch lengths on birth-death trees and the expected loss of phylogenetic diversity. *Syst. Biol.* 61: 195–203.

**Model Posterior Distributions**

Stem diversification rate vs. Genera extinction Risk


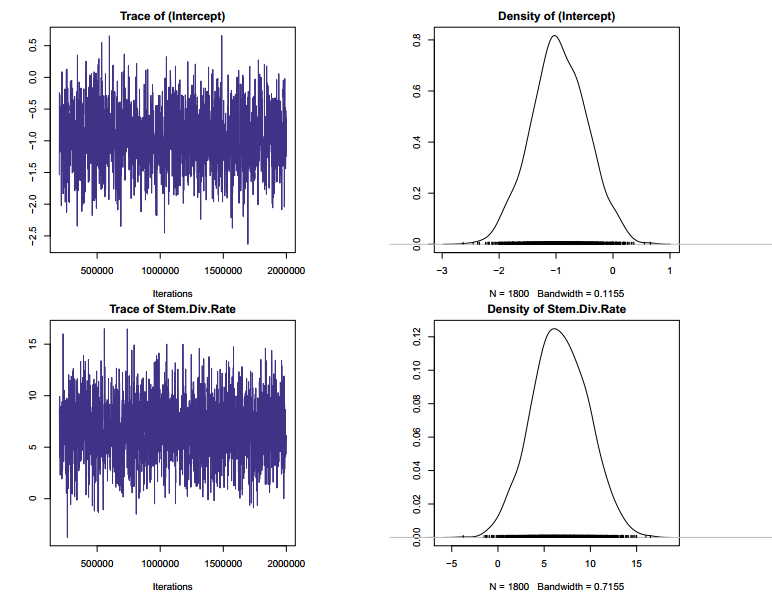


Crown diversification rate vs. Genera extinction Risk


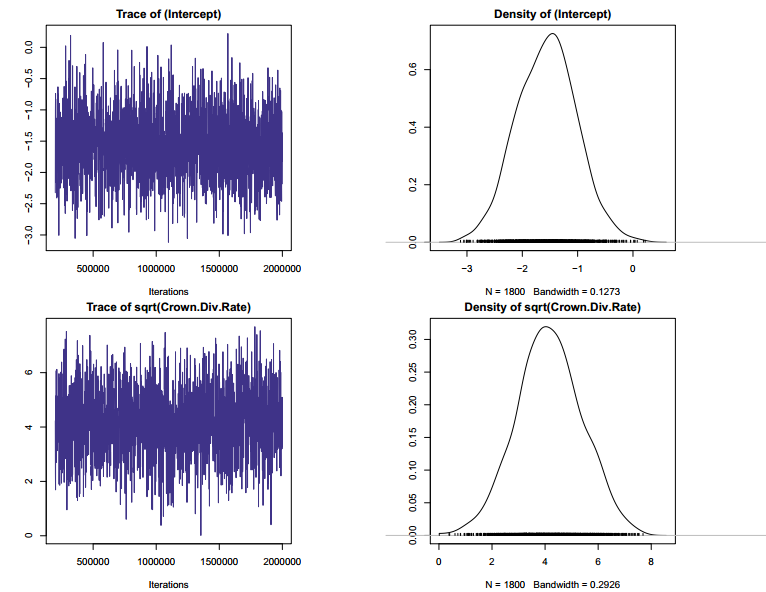


Stem diversification rate vs. Genera Range Size


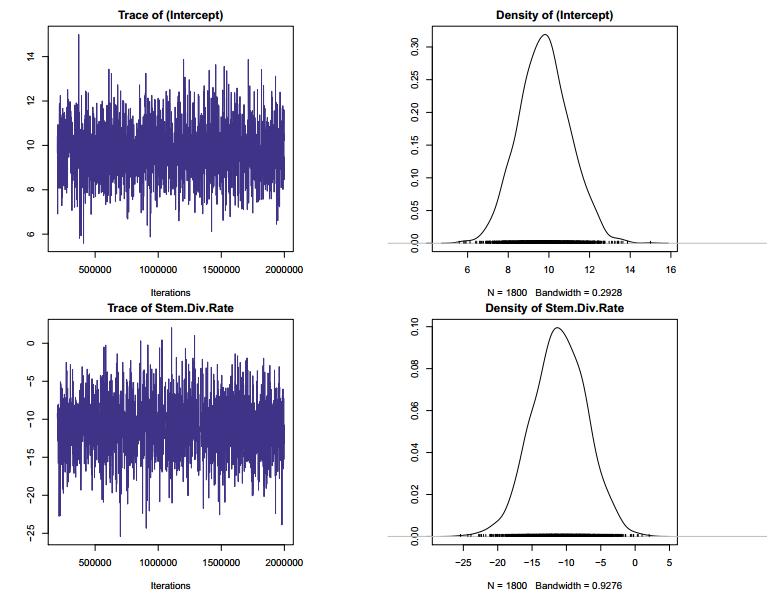


Crown diversification rate vs. Genera Range Size


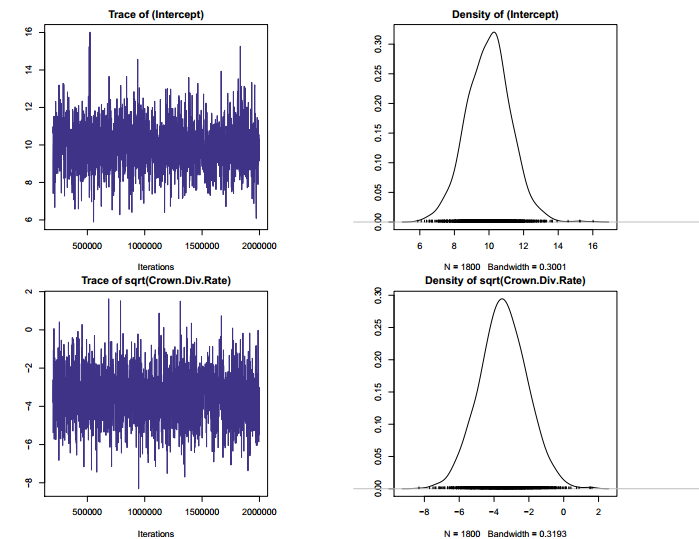

Supplement: Supplementary file 1 — Table S1. Twenty amphibian genera with all assessed species at risk, and therefore facing potential lineage extinction, indicating the species richness and phylogenetic diversity (PD) within each group, the lineage's stem age, and the potential prevention of PD loss by saving one species within each clade (PD saved). [file EVL3-1-40-s001.docx]
